# Effect of process conditions on the stability and physicochemical properties of green coffee oil Pickering emulsions stabilized with ZnO nanoparticles for quercetin-enhanced sunscreen formulations

**DOI:** 10.1186/s11671-026-04623-5

**Published:** 2026-05-18

**Authors:** César Uriel Rodríguez-Fuentes, Ana Guadalupe Castillo-Olmos, Julieta del Carmen Villalobos-Espinosa, Edgar Torres-Maravilla, Daniel Balleza, Zorba Josué Hernández-Estrada, Cynthia Cano-Sarmiento

**Affiliations:** 1 Unidad de Investigación y Desarrollo en Alimentos, Tecnológico Nacional de México/ Instituto Tecnológico de Veracruz, M.A. de Quevedo 2779, 91897 Veracruz, México; 2 Ingeniería en Industrias Alimentarias, Tecnológico Nacional de México/ITS de Teziutlán, Fracción I y II Aire Libre S/N, 73960 Teziutlán, Puebla México; 3 Facultad de Medicina Mexicali, Universidad Autónoma de Baja California, 21000 Mexicali, México

**Keywords:** Pickering emulsion, Sunscreen, Quercetin, Rheological behavior, Turbiscan stability index

## Abstract

Solar ultraviolet (UV) radiation is the primary etiological factor in the development of several cutaneous malignancies, including carcinomas. In this context, the use of sunscreen formulations usually helps to prevent and reduce UV skin damage. The aim of this work was to explore the impact of the process conditions on some relevant physicochemical properties in sunscreens with a formulation based on quercetin Pickering emulsions stabilized with ZnO particles. Four formulations were prepared by controlling the speed and homogenization time using green coffee oil as the external phase and a mixture of stabilizers, water, and polyethylene glycol as the dissolution media. The stability of the emulsified systems was analyzed in terms of time after 28 days of storage by optical microscopy and digital image analysis to determine the mean particle size. The Turbiscan Stability Index (TSI), Sun Protection Factor (SPF), rheological behavior, and antioxidant activity were also evaluated. The system with the highest physical stability, minimal changes in rheological properties, and superior stability during storage time with respect to breakage phenomena was obtained at 15,000 rpm/2 min, with a SPF of ~ 40 and exhibiting one of the highest antioxidant capacities compared to other treatments. This stability was constant during the evaluation period. With these results we established the optimal conditions for the potential development of sunscreens with desirable attributes to reduce the harmful effects of UV radiation in addition to establishing the encapsulation conditions of bioactive compounds and facilitating its scale-up.

## Introduction

Exposure to ultraviolet (UV) radiation from sunlight is a critical risk factor in skin carcinogenesis [56]. In that sense, the use of sunscreen is essential for preventing and reducing UV damage such as burns, photoaging, and abnormal pigmentation [16, 59]. Sunscreens exert their protective effects through different mechanisms of action, including: (i) acting as a chemical barrier, by including compounds capable of absorbing damaging wavelengths, and (ii) set up a physical barrier to reflect and disperse potentially dangerous light [50, 65]. Most sunscreen formulations are primarily composed of synthetic drugs exerting their protective effects against photoirritation, photosensitization, and dermatitis [28, 75]. However, the development of formulations that include biocompatible compounds of natural origin is a desirable attribute in the constant search for strategies to protect human health from the harmful effects of UV radiation.

Several research groups have conducted investigations on the protective effects of plant oils present in flowers, olives, coconut, green coffee, and other plants [5, 31, 43, 52, 54, 55, 83, 95]). Among these natural sources, green coffee oil stands out for its sun protection factor of ~ 5, along with antioxidant and moisture-retaining properties. This natural oil is characterized by being a complex mixture of antioxidant compounds with a high concentration of triacylglycerides, diterpenic esters, fatty acids, tocopherols, and diverse sterols, which act as UV radiation absorbers [63, 84, 94, 95]. Incorporating this kind of natural oil into a cosmetic dermal product has the potential to create a matrix that transports antioxidant compounds [5, 54, 94]. The sun protection products include creams, sprays, lotions, gels, and emulsions. Emulsions have been widely used due to their properties for enhancing the bioactivity of diverse chemical compounds and achieving stable systems with better compound distribution throughout the product, thus facilitating the incorporation of bioactive compounds with photoprotective activity [28]. Pickering emulsions are colloidal dispersions of two immiscible liquids that are stabilized through the incorporation of solid particles or nanoparticles in the interfacial region, forming a protective barrier around the droplets. This structure provides greater rigidity and reduces the contact area between the phases [40, 104]. Remarkably, the key property of this nanotechnology lies in the fine control of the degree of wetting of the nanoparticles at the oil-water interface, which reduces their interfacial free energy and enhances emulsion stability [92]. However, to functionalize such a system, strict control over conditions of their formation is crucial, as these depend on several parameters, including particle size, distribution, and stability [33, 101].

Pickering emulsions, also known as surfactant-free emulsions, utilize solid nanoparticles that can serve a dual function: as emulsifiers and as physical barriers. Thus, they allow the reduction in the use of synthetic components [6]. Nevertheless, typical sunscreen formulations, composed of Pickering emulsions, utilize micrometer-scale particle sizes, which affect the rheological properties of the product during application, resulting in a white cast upon skin deposition [2, 6, 33]. In contrast, nanoparticles with a minimum particle size of 100 nm avoid skin permeation, improve the product’s appearance after application, and enhance their optical properties, such as the ability to refract incident light [48, 81]. Additionally, from a rheological perspective, nanoparticles provide stability and structure to the product without compromising its spreadability on the skin [45, 53]. Among the solid particles used in sunscreen formulations, TiO_2_, ZnO, and aluminum starch are widely used. Due to the low toxicity of nanoparticles and their high efficacy in protecting the skin, ZnO particles have been widely preferred in the formulation of diverse sunscreen products in combination with other nanomaterials, such as fatty acids, silica derivatives, and polymers, which reduce or eliminate the potential production of free radicals and improve adherence to the skin, preventing its permeability [41, 45, 48, 81].

Emulsions enable the encapsulation of a wide range of natural compounds, such as flavonoids, a group of plant phenolic derivatives with potential nutraceutical, pharmaceutical, medicinal, and cosmetic applications, including their addition to some sunscreen systems [68]. Quercetin (2-(3,4-dihydroxyphenyl)-3,5,7-trihydroxychromen-4-one) is a flavonoid with anti-inflammatory, anticarcinogenic, and antioxidant properties. Additionally, it can increase the sun protection factor (SPF) when combined with physical barriers [13]. However, quercetin and other bioactive compounds are prone to degradation, exhibiting relatively low bioavailability. Hence, nanosystems are attractive for protecting those labile chemical compounds as well as optimizing their absorption and biological activities [24, 25]. Hatahet et al., [36] reported that an acceptable performance of substances in terms of activity could be achieved by using lipid nanosystems, water in oil (W/O) micro- and nanoemulsions, or collagen matrices. The use of W/O Pickering emulsions not only increases the bioavailability of quercetin but also allows the creation of a stable system in which inorganic compounds can also be introduced to enhance the functionality of the final product [33]. Green coffee oil has been reported as a carrier for compounds like melatonin (5-methoxy-*N*-acetyltryptamine). However, changing the bioactive compound can affect the stability and functional properties of the final product, making each evaluation specific to the molecular cargo. Furthermore, although Pickering emulsions have been studied for sunscreen applications and quercetin encapsulation separately, limited information exists on systems where ZnO particles simultaneously act as sunscreen agents and Pickering stabilizersto encapsulate quercetin using green coffee oil as the carrier phase.

Due to the influence of the formation process in emulsion systems on the encapsulation of bioactive compounds, the aim of this study was to evaluate the effect of Pickering emulsion formation and stabilization process conditions on key physicochemical properties of sunscreens, including quercetin as cargo and ZnO nanoparticles as molecular stabilizers.

## Materials and methods

### Materials

All reagents and solvents used were of analytical or HPLC grade. Green coffee oil was purchased from CREACOS (Guadalajara, Mexico). Zinc oxide nano coated with triethoxycaprylylsilane (ZnO) (Z Cote^®^ HP1) was purchased from BASF (Ludwigshafen, Germany), glycerol and polyethylene glycol (PEG) from Sigma-Aldrich^®^ (St. Louis, MO, USA), and xanthan gum from Albert and Ferran Adrià^®^ (Barcelona, Spain). The bioactive compound used was Quercetin ≥ 95% (Sigma-Aldrich^®^, St. Louis, MO, USA).

### Characterization of ZnO nanoparticles

#### Particle size distribution, morphology and optical properties

The morphology of ZnO nanoparticles was examined using a Tescan^®^ MIRA3 scanning electron microscope (Brno, Czech Republic) operated at 10.0 kV in high-vacuum mode with a secondary electron detector at a working distance of 9.39 mm. The sample was mounted on carbon tape, and the obtained micrographs were processed using MIRA3 software version 4.2.19.1 (Tescan^®^, Brno, Czech Republic) in TIFF format.

Particle size was determined using dynamic light scattering (DLS) on a Zetasizer Nano ZS equipment (Malvern Panalytical, UK), following the methodology of Assis-Dias-Alves et al., [4]. The nanoparticles were suspended in ethyl acetate to obtain a final concentration of 0.2 mg/mL and subjected to ultrasonic treatment for 1 h. Subsequently, 1 mL of the suspension was transferred to a glass cuvette for measurement.

The optical properties were evaluated through the absorption spectrum of ZnO nanoparticles coated with triethoxycaprylylsilane dispersed in ethyl acetate, obtained using a UV–Vis spectrophotometer (UV–Vis Agilent 8453, Agilent Technologies, Santa Clara, USA) in the range of 200 to 600 nm. The same nanoparticle concentration as used for size analysis was employed (0.2 mg/mL).

#### Contact angle determination

For the determination of the contact angle, the glass slide methodology was used with modifications [64]. To prepare the surface covered with nanoparticles, double-sided tape was placed on a microscope slide, followed by the deposition of a uniform layer of ZnO nanoparticles. This layer was lightly compressed to ensure material fixation, with excess particles removed. With the prepared surface, 5 µL of distilled water was placed on the nanoparticles using a 26 S syringe. Subsequently, a digital microscope (Celestron Handheld Digital Microscope Pro, Celestron LLC, CA, USA) was used to capture the micrograph, utilizing Celestron Microcapture Pro 2.5 software (Celestron LLC, CA, USA). The micrographs were saved in JPG format and processed using digital image analysis following the method reported by Stalder et al., [85].

For digital image analysis, ImageJ software version 1.53t (NIH, USA) was employed, utilizing the drop analysis plugin with the LB-ASDA function. Parameters were adjusted so that the contour outlined by the software matched the droplet’s contour in the image, and so the contact angle was obtained. The procedure was performed in triplicate with different samples, and the results are presented as mean ± SD.

### Preparation of emulsions

Emulsions were prepared according to the procedure described by Marto et al., [54], with modifications in the dispersed phase and the type of nanoparticles used. Green coffee oil was selected for its ability to solubilize quercetin and its compatibility with topical use, while ZnO particles were used due to their dual function as Pickering stabilizers and UV-blocking agents. The ingredient selection and proportions were carefully defined to ensure emulsion stability and effective encapsulation of bioactive compounds. The final composition is shown in Table 1. In compliance with Regulation 1223/2009 of the European Parliament and Council, the INCI names and the function of the ingredients in the formulation are listed alongside their respective percentages.

**Table 1:** Composition of Pickering emulsion sunscreen

Component (INCI Name)	Function	Quantitative composition (%, w/w)
*Continuous phase*		69.64
*Coffea Arabica* (coffee) seed oil	Anti-aging agent / Antioxidant / Emollient / Moisturizing agent	46.12
Zinc Oxide (and) Triethoxycaprylylsilane	UV filter / Skin protector	23.52
*Disperse phase*		30.36
Polyethylene glycol	Solvent / Emulsion stabilizer	10.00
Glycerin	Humectant / Viscosity controller	6.65
Aqua	Solvent	13.36
Xanthan gum	Gel forming / Viscosity controller / Emulsion stabilizer	0.30
Quercetin	Antioxidant	0.05

Zinc oxide and green coffee oil were homogenized for the continuous phase using magnetic stirring PC-420D (Corning^®^, NY, USA) at 1100 rpm for 30 min. Then, ZnO was dispersed with higher energy using a rotor-stator homogenizer Ultraturrax^®^ T25 (IKA^®^, Staufen, Germany) at 13,000 rpm for 1 min to ensure that most particles came into contact with the oil phase [6].

The dispersed phase was prepared by dissolving the bioactive compound in PEG using an ultrasonic bath Aquawave 9376^®^ (1.75 L, 115 V, 50/60 HZ, 500 W) (Barnstead Lab-Line, IA, USA) at room temperature (25 °C) for 5 min. Next, purified water, xanthan gum, and glycerol, which had been previously mixed manually, were added.

Phases were mixed at the phase proportion previously defined in Table 1 using a rotor-stator homogenizer while varying the working conditions in terms of time (min) and speed (rpm) according to Table 2. Each cycle consisted of 1 min of work followed by 1 min of rest. The obtained emulsions exhibited a pH ranging from 6 to 6.5 and were physically characterized. The effect of the processing conditions on their physicochemical properties was evaluated. Additionally, their potential for encapsulating bioactive compounds was assessed to develop a system that could be considered functional as a sunscreen.

**Table 2:** Operating conditions for Pickering emulsion sunscreen

Treatment	Speed (rpm)	Time (min)
S13T2	13,000	2
S13T4	13,000	4
S15T2	15,000	2
S15T4	15,000	4

Equipment used for emulsification was a high shear rotor – stator homogenizer Ultraturrax^®^ T25

The analyses were conducted on the emulsions both on the preparation day and after 28 days of storage in dark conditions at 25 °C and 36.5% relative humidity, using a crystal cell.

### Particle size distribution

Particle size distribution was assessed using a DM2000 LED optical microscope (Leica Microsystems^®^, Wetzlar, Germany) at 100$\:\times\:$ magnification. Micrographs were captured using Leica Application Suite V4.9 software. Each sample was prepared by dispersing 0.25 g of each emulsion in 1 mL of green coffee oil, and 5 µL of the final dispersion was placed on a glass slide. The micrographs were saved in TIFF format using the automatic settings provided by the software and subsequently analyzed through digital image analysis. Emulsions were evaluated at both 0 and 28 days after storage [3, 69].

### Digital image analysis

**Fig. 1:**
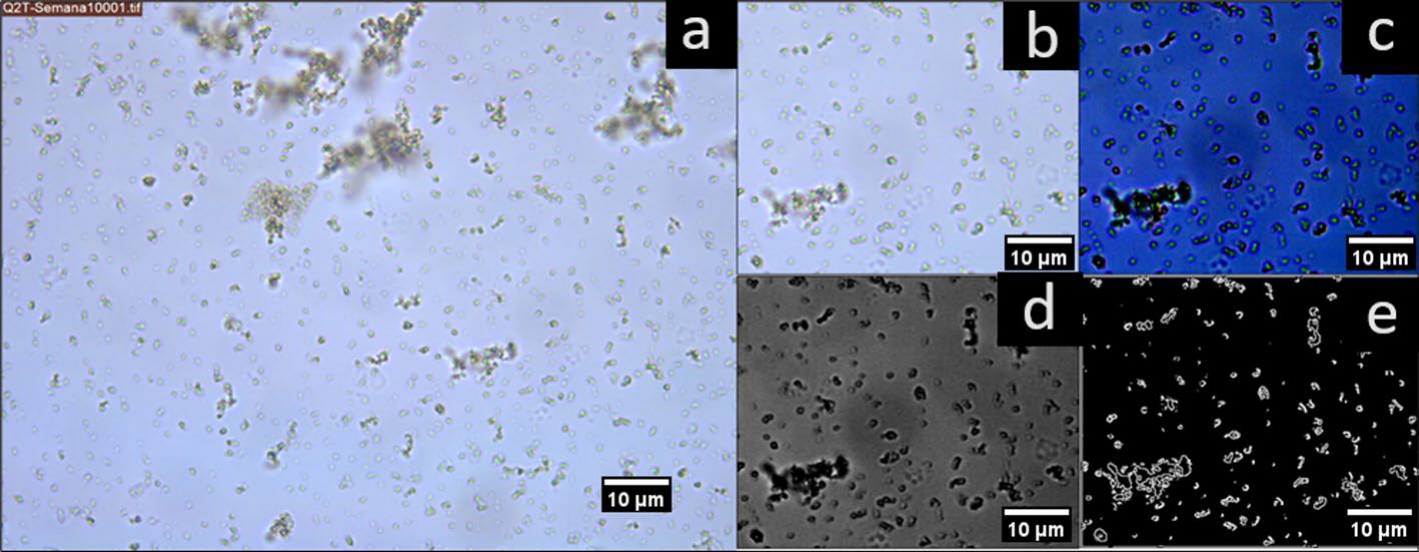
**a** Typical micrograph taken from the emulsion droplets and its processing for digital analysis. **b** Crop of selected area. **c** Contrast modification. **d** Binarization. **e** Outlined particles

The average particle size D[3:2] and Feret diameter were carried out using ImageJ 1.53t software (NIH, USA), following previous reports by Cano-Sarmiento et al., [7] with some modifications. The contrast and brightness of the micrographs were manually adjusted to ensure optimal visualization of the particles. Subsequently, the images were converted to 8-bit format, and the threshold level was automatically adjusted to generate a binary image. The *Binary: Outline* function was used to outline the contours of the particles, and finally, the *Analyze Particles* option was applied. For all treatments, at least three areas per image were analyzed at both 0 and 28 days of storage. Figure 1 illustrates the steps and modifications in the micrographs for digital image analysis, from which the average particle size was estimated.

### Physical stability of emulsions

The Turbiscan Stability Index (TSI) was measured to assess the presence of instability phenomena. The TSI value was determined following the methodology described by Li et al., [46] with a Turbiscan Lab^®^ Expert instrument (Formulaction, Toulouse, France). It was performed through multiple light scattering technique, where a sample of each emulsion was placed in cylindrical glass cells. The measurements were conducted at 25 °C for 28 days. TSI values close to zero indicate higher emulsion stability [42].

### Rheological characterization

Pickering emulsions were analyzed for rheological behavior using a rheometer MCR 92 and RheoCompass 1.30 software (Anton Paar, Graz, Austria) with a 25 mm diameter plate-plate geometry equipped with a Peltier temperature control system set at 25 °C, and a gap of 0.5 mm between each plate.

#### Rotational test

Emulsions were subjected to flow curve tests over a shear rate range of 0.1 to 100 1/s, and shear stress vs. shear rate data were plotted for each treatment. These data were then fitted to the Herschel-Bulkley (HB) model (Eq. 1) to characterize the behavior and structural changes that occurred in the emulsion at time zero and after 28 days of storage [8, 11, 47, 78].

1 $$\:\sigma\:={\sigma\:}_{0}+k\cdot\:{\gamma\:}^{n}$$

where $\:\sigma\:$ represents the shear stress (Pa), $\:{\sigma\:}_{0}$ is the yield stress (Pa), $\:k$ is the consistency index $\:\left(\mathrm{P}\mathrm{a}\cdot\:{\mathrm{s}}^{n}\right)$, $\:\gamma\:$ is the shear rate (s^− 1^), and $\:n$ is the flow behavior index (dimensionless).

#### Oscillatory test

Amplitude sweeps were conducted for each treatment over a shear strain range of 0.01 to 100% at a constant angular frequency of 10 rad/s to determine the linear viscoelastic region (LVR). This region is characterized by constant storage $G^{\prime}$ and loss moduli $G^{\prime\prime}$ [15]. From the LVR, a specific strain value was selected and fixed in frequency sweep tests to analyze the structural changes inherent to the system, rather than those induced by equipment-generated stresses [29]. Additionally, a variation in angular frequency from 1 to 100 rad/s was performed to evaluate $G^{\prime}$ and $G^{\prime\prime}$ relate to the conformation of the emulsion structure and the product’s consistency characteristics [49].

### In vitro sun protection factor

The Sun Protection Factor (SPF) was determined using the standards proposed by Ahmad-Zaki et al., [2] for sunscreens containing ZnO nanoparticles. One gram of the emulsion was dissolved and made up to 100 mL with 40% ethanol. The solution was subjected to an ultrasonic bath Aquawave 9376 (1.75 L, 115 V, 50/60 HZ, 500 W, Barnstead Lab-Line, IA, USA) for 5 min at 25 °C and filtered through cotton, discarding the first 10 mL. An aliquot of 5 mL was diluted and made up to 50 mL with the same solvent, resulting in a solution with a concentration of 1 mg/mL. The same procedure was done with the commercial 25% ZnO 50 SPF labeled sunscreen as a reference.

The absorbance was measured using a UV–Vis spectrophotometer Agilent 8453 (Agilent Technologies, CA, USA) with a 1 cm glass cuvette and 40% ethanol as the blank. Data were obtained at wavelengths from 290 to 320 nm, in 5 nm increments, and were substituted into Eq. 2 [51] to determine the SPF value.

2 $$\:\mathrm{S}\mathrm{P}\mathrm{F}=\mathrm{C}\mathrm{F}\mathrm{*}{\sum\:}_{320}^{290}\mathrm{E}\mathrm{E}\left({\uplambda\:}\right)\mathrm{*}\mathrm{I}\left({\uplambda\:}\right)\mathrm{*}\mathrm{A}\mathrm{b}\mathrm{s}\left({\uplambda\:}\right)$$

where $\:\lambda\:$ represents the wavelength, $\:EE\left(\lambda\:\right)$ is the erythema effect spectrum, $\:I\left(\lambda\:\right)$ is the solar spectrum’s intensity, $\:Abs\left(\lambda\:\right)$ is the sunscreen’s absorbance, $\:CF$ is a correction factor (10), and $\:SPF$ is the sun protection factor. This equation is a correlation between the absorbance at each wavelength and the equivalent to the intensity at the same wavelength using constant values of *EE* x *I* reported elsewhere [74].

### Inhibition of ABTS^•+^ radicals

The free radical ABTS^**•+**^ (2,2′-azino-bis(3-ethylbenzothiazoline-6-sulfonate) solution was prepared according to Espino-Manzano et al., [21]. Separate solutions of 7 mM ABTS^**•+**^ and 2.45 mM potassium persulfate (K_2_S_2_O_8_) were prepared in distilled water, mixed in a 0.5:1 ratio, and stored at 25 °C in the dark for 12 h. Subsequently, the resulting solution was diluted with ethanol to achieve an absorbance of 0.700 ± 0.02 at $\:\lambda\:=734$ nm. Sample preparation was carried out using the procedure described for SPF evaluation, with a final concentration of 1 mg/mL of the emulsion in 40% ethanol. The samples were stored in amber vials at room temperature (25 °C) to prevent compound degradation due to light exposure.

The method of Medina-Pérez et al., [58] was used to evaluate the antioxidant activity, with modifications in the volume of ABTS^**•+**^ used. Briefly, 200 µL of the sample was added to 1800 µL of the ABTS^**•+**^ radical solution and incubated in the dark at room temperature (25 °C) for 6 min. The same procedure was carried out with 40% ethanol to obtain a blank. Absorbance was measured at 734 nm using a UV–Vis spectrophotometer Agilent 8453 (Agilent Technologies, CA, USA), and the obtained data were substituted into Eq. 3 [21] to determine the percentage of free radical inhibition.

3 $$\:Inhibition\:\%=\left(\frac{{A}_{0}-{A}_{A}}{{A}_{0}}\right)*100$$

where $\:{A}_{0}$ is the absorbance value of the blank, and $\:{A}_{A}$ is the absorbance value of the sample.

### Statistical analysis

Statistical analysis of the obtained results was conducted using analysis of variance (ANOVA) and Tukey’s mean comparison with Graph Pad Prism^®^ 8 software (GraphPad Software, Inc., San Diego, CA). Curve fitting was performed using OriginLab 2021 (9.8.0.200) software (OriginLab Corporation, Northampton, Massachusetts). A significant alpha level of *p* < 0.05 was used (*n* = 3).

## Results and discussion

### Characterization of ZnO nanoparticles

Zinc oxide nanoparticles are widely used in the formulation of inorganic physical sunscreens due to their superior protective properties compared to organic compounds. Additionally, they offer advantages such as the absence of skin irritation or sensitivity, low skin penetration, and broad-spectrum coverage. However, as particle diameter decreases, the surface-to-volume ratio increases. Consequently, nanoparticles may exhibit higher reactivity than traditional materials, enhancing their utility in biomedical applications but also increasing the risk of potential health and environmental hazards [86]. In this context, it is crucial to understand their characterization in terms of size, morphology, contact angle, and optical properties before incorporating them into final sunscreen products [44, 81]. Coatings such as triethoxycaprylylsilane and silica derivatives have been employed to reduce ion generation and migration from the material to the skin [41, 70]. However, these nanofunctionalized particles can alter important physical characteristics, including morphology, particle size, and contact angle, thereby modifying the properties of the formulations obtained.

**Fig. 2:**
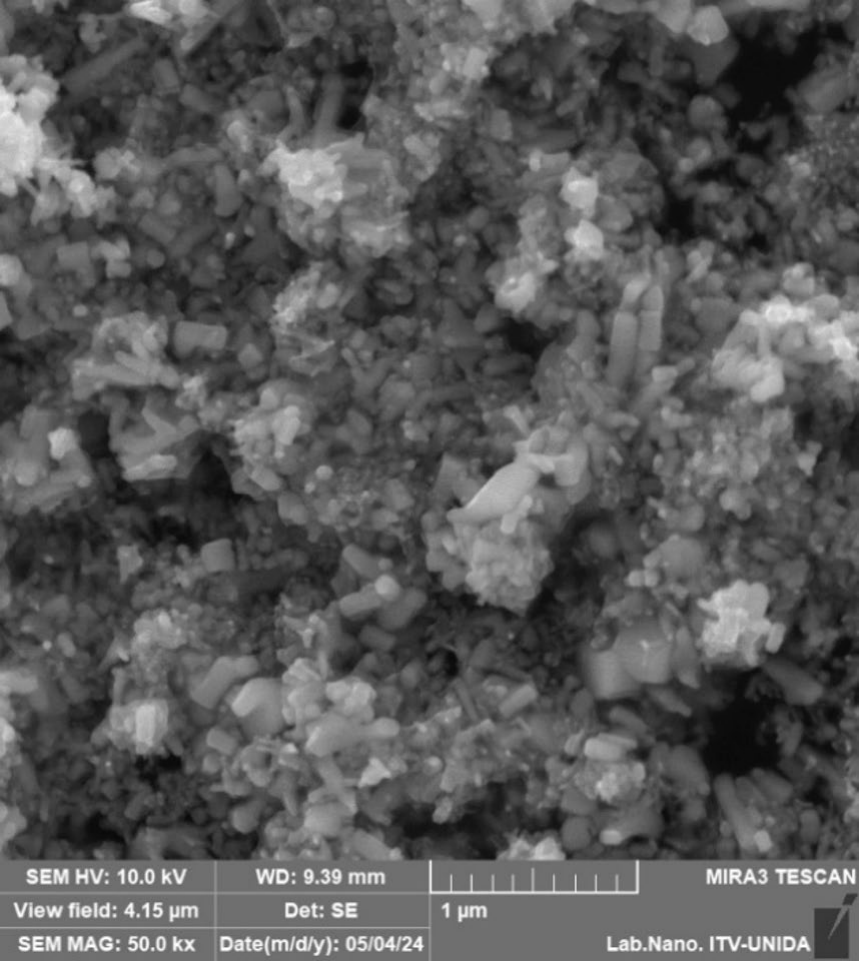
SEM micrograph of zinc oxide coated with triethoxycaprylylsilane (Z-COTE HP1, BASF^®^)

The zinc oxide exhibits multiple morphologies, with the most commonly observed being rod-shaped (Fig. 2). This observation aligns with the information provided by the supplier in their technical datasheet. Furthermore, dynamic light scattering (DLS) analysis revealed an average particle size of 134.8 ± 1.2 nm and a D90 percentile of 243 ± 17.4 nm. Regulatory definitions classify nanomaterials as materials with particle sizes between 1 and 100 nm. In topical cosmetic applications, although nanomaterials are typically defined within this range, particles exceeding this threshold are often selected to avoid regulatory constraints associated with nanomaterial classification and labeling. In this context, the ZnO particles used in this study (> 100 nm) are consistent with their intended topical application and are expected to exhibit minimal dermal penetration and limited systemic absorption under typical conditions of use as reported by Hansen et al., [34].

**Fig. 3:**
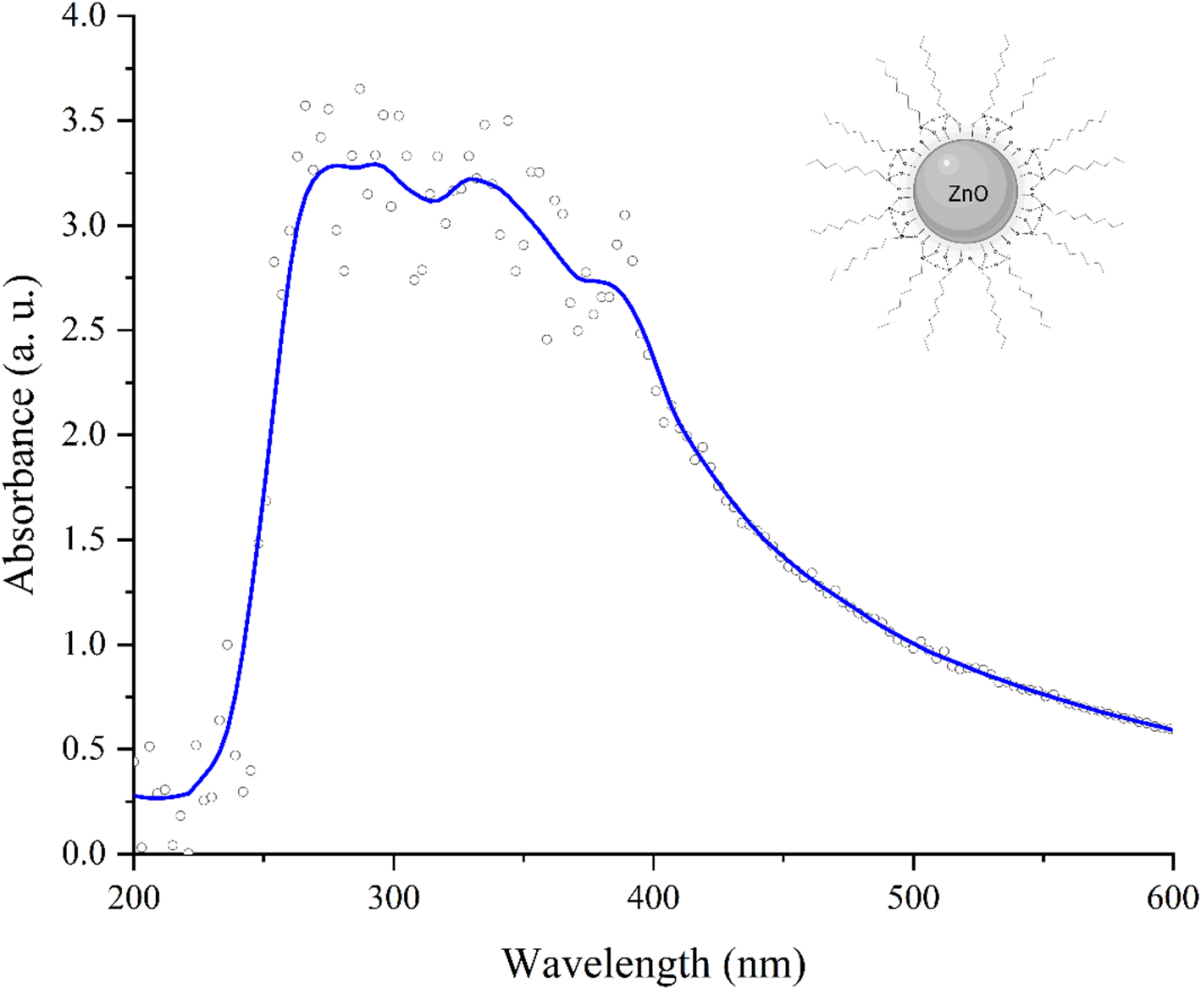
UV–Vis absorbance spectra of ZnO coated with triethoxycaprylylsilane

The advantages of using nanoscale zinc oxide in cosmetic formulations, such as sunscreens, include its enhanced surface area and intrinsic properties, such as a high refractive index and absorption in the UV-A (315**–**400 nm) and UV-B (280**–**315 nm) regions [44, 82]. As shown in Fig. 3, ZnO coated with triethoxycaprylylsilane exhibits strong absorption from 250 to 400 nm. In comparison, bulk ZnO has a maximum absorption at 375 nm. Due to its nanoscale size, coating, and multiple morphologies, a shift and broadening of this absorption range are observed, which can be beneficial, as it indicates enhanced photoprotective activity through increased absorption across the UV spectrum [1, 79].

### Contact angle

In both conventional and Pickering emulsions, the region between the two liquid phases contains a mixture of oil, water, and emulsifying agents (i.e., solid particles or surfactant molecules). The properties of this region determine the emulsion type (W/O or O/W), with wettability being crucial for particle-stabilized systems. Wettability is determined by the contact angle [100], which indicates the affinity of particles for one of the two phases. According to Finkle’s empirical rule, particles with contact angles below 90° are predominantly hydrophilic and stabilize O/W emulsions, whereas angles greater than 90° are more hydrophobic and stabilize W/O emulsions [32, 72]. In this study, ZnO particles coated with triethoxycaprylylsilane showed a contact angle of 121.6 ± 1.6°, indicating a stronger tendency to form emulsions with a dispersed aqueous phase. This behavior is attributed to the coating, which contains polar molecules with some affinity for oil. This characteristic is advantageous for developing topical products, as a continuous oil phase exhibits greater affinity and adhesion to the skin, enhancing its water resistance and prolonging its retention on the skin [4, 54].

### Particle size distribution

In emulsion studies, several reports highlight the influence of process parameters, such as speed, temperature, and processing time, on particle size and their stability [87, 89, 91]. Specifically, speed influences droplet breakage and size reduction, with high speeds imparting significant energy that can lead to smaller sizes and greater homogeneity, albeit at the expense of temperature rises that may degrade organic compounds such as quercetin [9, 35, 102]. Processing time is crucial for Pickering emulsions, as it facilitates proper component contact and, with longer times, allows better particle arrangement around the continuous phase [35]. Temperature also influences emulsion systems, as elevated temperatures can promote droplet mobility and destabilization phenomena. Although Pickering emulsions exhibit enhanced structural stability due to particle adsorption at the interface, where they form a barrier that confers steric resistance, increased temperatures may still induce degradation of thermosensitive bioactive compounds such as quercetin during processing [77].

In topical applications, particles larger than 100 nm are generally preferred, as they exhibit reduced potential for skin penetration and may avoid additional regulatory and toxicological requirements, including genotoxicity assessment. Consequently, detailed characterization of emulsion particle size is essential to ensure compliance with safety considerations [42].

In all samples analyzed in this study, the mean particle size D[3:2] was greater than 100 nm (0.44–0.53 μm) at day zero. After 28 days of storage, increases of 19%, 21%, 20%, and 11% were observed in the mean particle size for samples S13T2, S13T4, S15T2, and S15T4, respectively (Table 3). Meanwhile, percentile D90 shows a global increase of around ~ 0.1 μm at the same evaluated time.

**Table 3:** Mean particle size D[3:2] and percentile D90 of Pickering emulsions*

Treatment	Day 0		Day 28	
	D[3:2] (µm)	D90 (µm)	D[3:2] (µm)	D90 (µm)
S13T2	0.67 ± 0.08ª^A^	0.81 ± 0.12ª^A^	0.80 ± 0.06^aA^	0.95 ± 0.06^aA^
S13T4	0.65 ± 0.04ª^A^	0.83 ± 0.03ª^A^	0.79 ± 0.04^bA^	0.94 ± 0.08^aA^
S15T2	0.67 ± 0.08ª^A^	0.84 ± 0.09ª^A^	0.81 ± 0.06^aA^	0.91 ± 0.04^aA^
S15T4	0.69 ± 0.08ª^A^	0.89 ± 0.14ª^A^	0.77 ± 0.05^aA^	0.87 ± 0.04^bA^

*: Averages ± standard deviation (*p* < 0.05). Equal letters (upper at the same time, and lower for the same treatment) indicate no significant difference

**Fig. 4:**
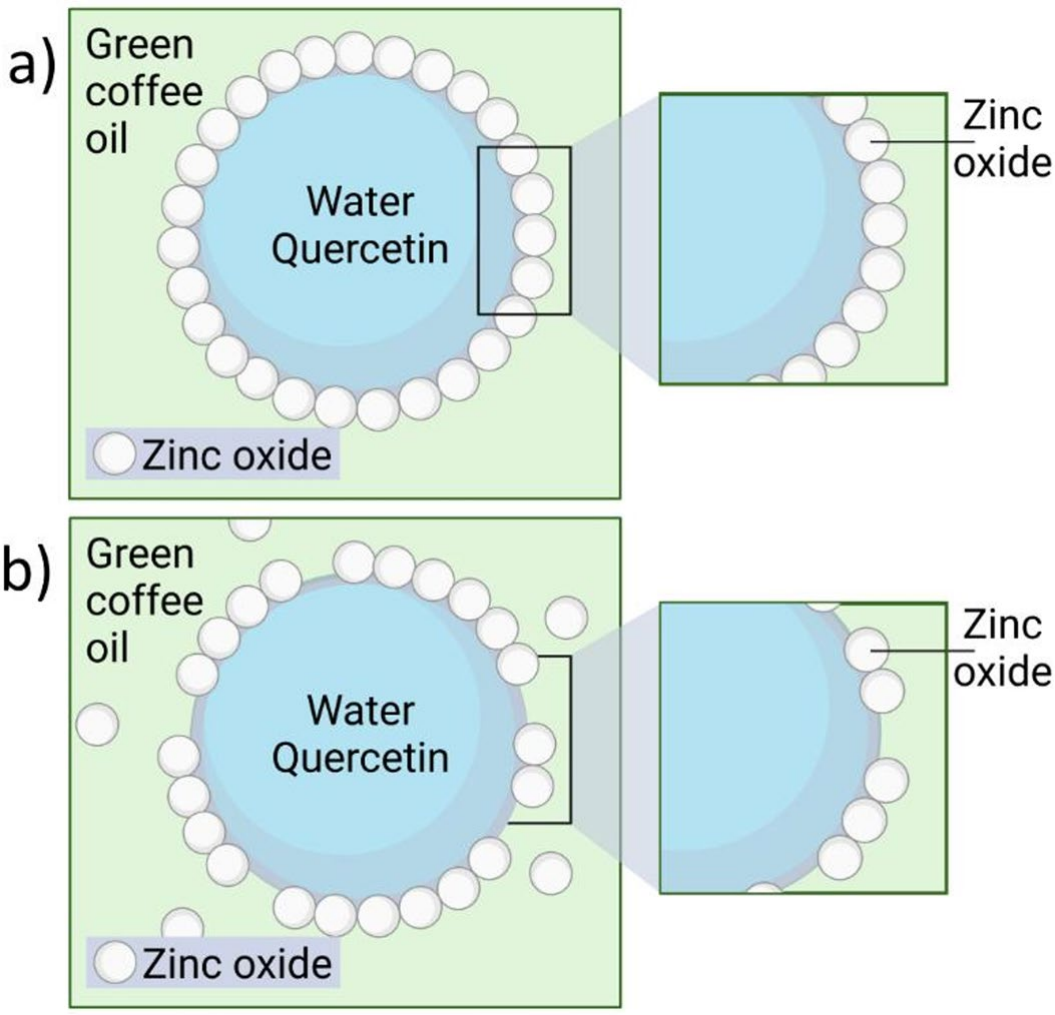
Schematic representation of emulsion components and their distribution in droplet conformation: (a) Desirable formation of an interfacial coarse. (b) Deficient, non-complete interfacial coverage

For Pickering emulsions, some authors [9, 33, 104] indicate that once the coating layer around the dispersed phase is formed correctly, it is challenging to break owing to the fact that the contact area between the two liquids has been reduced or even eliminated, and the water-particle-oil contact replaces the direct water-oil contact. Additionally, the material’s configuration can enhance stability and interactions with the continuous phase. This is because systems that are less prone to coalescence are produced. However, the coating may have areas of poor coverage due to uneven distribution of the interface [17, 104]. Even so, this possibility is minimized by the nanometer scale of the solid nanoparticles, which regulate droplet size and arrangement while reducing potential pores or oil-water contact areas where instability might occur (Fig. 4).

Although emulsions were prepared under different operating conditions, the treatments did not show a significant difference (*p* > 0.05) at both the initial (day 0) and final time (day 28) (Table 3). However, the particle size showed a trend toward increasing during storage, although the emulsion instability was not macroscopically visible. Furthermore, the optical microscopy analysis technique requires sample dilution, which can break up agglomerates or redistribute nanoparticles. Overcoming these limitations requires the use of other techniques to evaluate the physical properties of these microsystems in suspension, such as multiple light scattering.

### Turbiscan stability index

**Fig. 5:**
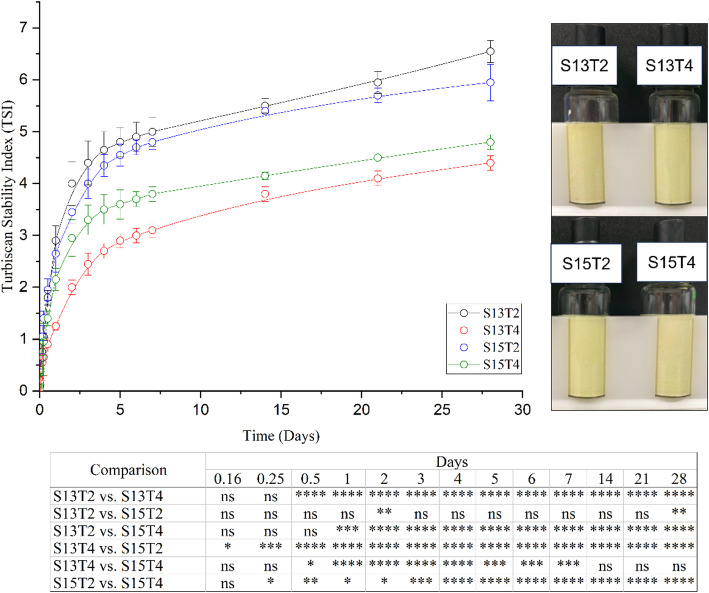
Turbiscan stability index (TSI) of Pickering emulsions elaborated by combinations of speed (13, 000–15,000 rpm) and time (2–4 min). TSI was measured for 28 days for storage stability. Two – way ANOVA test followed by Tukey´s multiple comparison. **** *p* < 0.0001. ***p < 0.001, **p < 0.01, *p < 0.05

Emulsions, being thermodynamically unstable systems, are prone to instability phenomena such as sedimentation, creaming, coalescence, flocculation, and Ostwald ripening, which can lead to phase separation [57]. The encapsulation of bioactive compounds is crucial for preserving the physicochemical properties of emulsions and ensuring the proper release and protection of compounds during storage [36]. Although Pickering emulsions are less susceptible to coalescence and Ostwald ripening, improper distribution of solid particles at the interface can promote instability phenomena [104]. Here, TSI was used to monitor and characterize the physical stability of the four emulsions over 28 days (Fig. 5). The TSI value is a parameter that quantifies the degree of instability in the emulsion by comparing backscattering values [103]. Since the composition remained constant, changes in TSI over time allowed observation of the effect of processing conditions on the development of a functional product.

Figure 5 depicts TSI values as a function of time for each treatment. Lower TSI values indicate higher emulsion stability, as they reflect reduced destabilization phenomena such as creaming, sedimentation, or coalescence during the evaluation period. Among the evaluated conditions, the treatment processed at 13,000 rpm for 4 min exhibited the lowest TSI values, indicating the highest stability based on turbidity measurements. No significant changes in emulsion stability were observed up to 4 h except for S13T4 vs. S15T2 (*p* < 0.05). As time progressed on day 2, significant differences in TSI values became evident among all treatments, with TSI values of 4.0, 2.0, 3.45, and 2.95 for S13T2, S13T4, S15T2, and S15T4 samples, respectively (*p* < 0.05). This gradual onset of differences can be attributed to the intrinsic properties of the emulsion, such as its viscosity and structural conformation, which can slow down the movement of droplets within the emulsion, thereby enhancing stability [73, 102]. From day 3 to day 21, no significant differences were observed between the TSI values of S13T2 and S15T2 (*p* > 0.05) (Fig. 5). To note, the S13T2 and S15T2 were generated at two different speeds, 13,000 rpm and 15,000 rpm, respectively. The change in mixing speed from 13,000 to 15,000 rpm did not significantly affect the emulsion behavior during short mixing times (2 min). However, at longer mixing times (4 min), this change in speed was decisive for the system’s behavior, suggesting that prolonged exposure enhances interactions between components, including van der Waals and electrostatic forces. These properties can contribute to either stability or instability in those systems, depending on the balance and control of materials selected during the formulation process [27, 57]. The speed parameter modulates the shear force in the homogenization zone, where higher speeds yield high shear forces; however, their use is limited by the associated temperature increase. At low speeds, both temperature increase and shear force are lower, therefore longer processing times must be used to compensate this effect; as shown in S13T2 treatment, where the low speed and short processing time affected the distribution of ZnO nanoparticles around the droplets, as a result, there was a higher presence of instability phenomena such as flocculation, coalescence, and Ostwald ripening, i.e. the thermodynamic process where small oil droplets are dispersed in an aqueous milieu ([27, 32, 62]). Regarding S15T4 treatment, a TSI value increase was observed compared to treatments S15T2 and S13T4, suggesting that the amount of energy required to form the emulsion was exceeded. The employed conditions resulted in a phenomenon referred to as over-processing, in which the energy density applied to the system is exceptionally high, favoring phenomena of coalescence due to the constant rupture and formation of droplets, along with a minor migration rate of nanoparticles to the interfacial region than the rate of their formation [67]. On day 28, both S13T4 and S15T4 treatments had the lowest TSI values, with final 4.4 and 4.8, respectively. Both treatments were more stable compared to others (6.5 and 5.9, TSI values for S13T2 and S15T2, respectively), even though they had the largest increase in particle size (57%) (Fig. 5). Overall, our results indicate that, in all treatments, the combination of speed and time indeed modifies the stability value.

### Rheological characterization

#### Flow curves

**Fig. 6:**
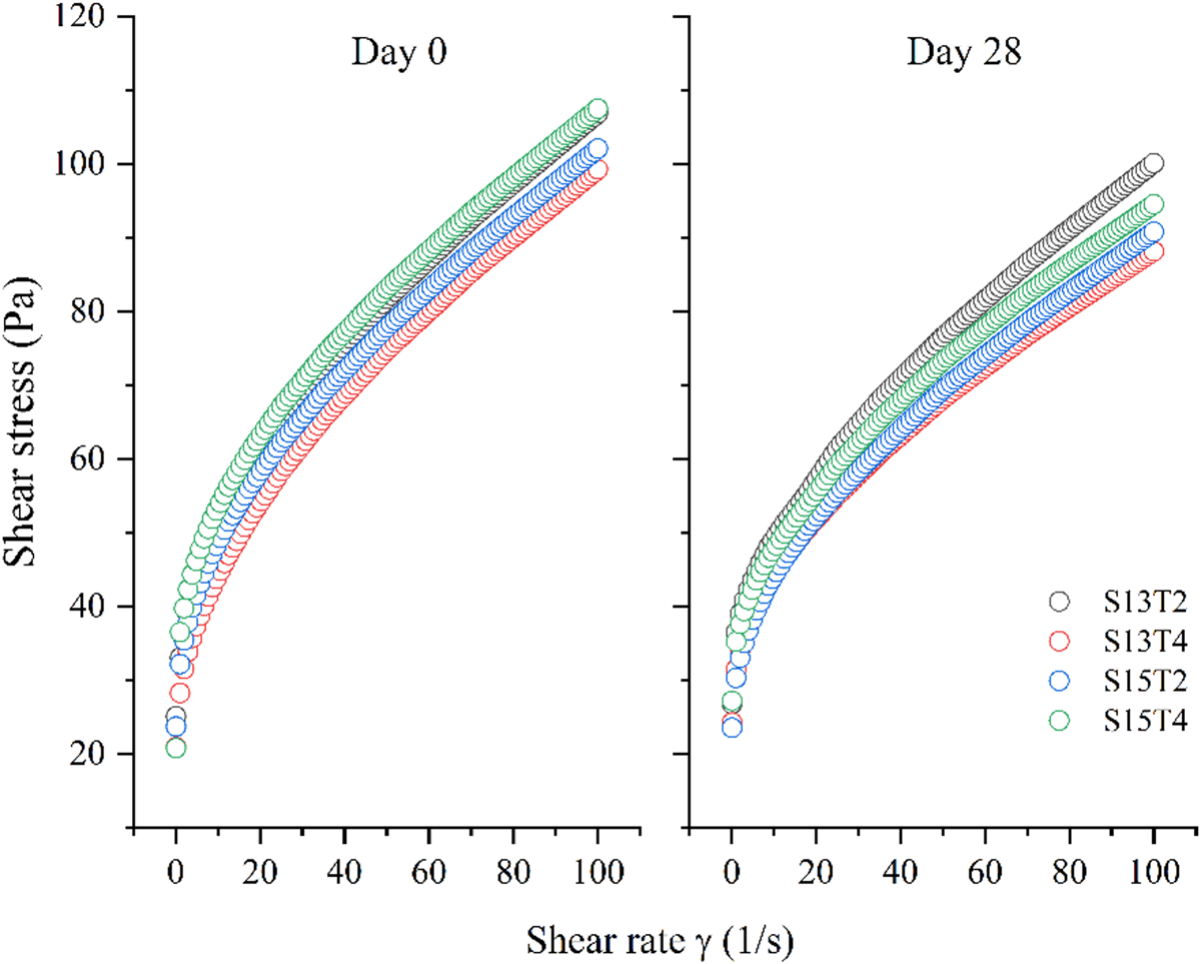
Flow curves (average, *n* = 3) of emulsions at days 0 and 28, showing shear-thinning behavior and shear rate dependence. Variations in yield stress and consistency are influenced by particle size and hydrophobicity

Characterizing the flow behavior of O/W emulsions enables the study of packing systems in relation to product filling, skin assimilation, and ease of product extraction [105]. The viscosity of these emulsions depends on shear rate, a typical characteristic of non-Newtonian fluids, as evidenced by the flow curves (Fig. 6). This behavior may be related to the hydrophobicity of the materials used. In this sense, Hohl et al., [38] suggested that the hydrophobicity of the particles, rather than their surface properties, has a greater influence on the stability of the emulsified structure. Additionally, flow curves help understand the physical behavior of fluids under stress. Given that for topically applied products, an excellent spreadability is a desirable attribute [33], all emulsions obtained in this work showed an acceptable shear-thinning behavior (Fig. 6). According to Bordes et al., [6] and Marto et al., [54], shear-thinning behavior enables better application and distribution on the skin, creating a homogeneous film that ensures proper deposition of particles. This enhances their UV protection capacity by forming a single zone for reflection and refraction of UV rays.

The emulsions data curve was fitted to the Herschel-Bulkley (HB) rheological model (*R*^2^ > 0.99) to obtain parameter values that allow a clear comparison and definition of rheological behavior [6]. This model represents the behavior of non-Newtonian fluids that exhibit a yield stress before flow begins, as is typical of thixotropic fluids, and is widely used in pharmaceutical and cosmetic products [37, 52]. Table 4 depicts the obtained values.

**Table 4:** The Herschel- Buckley model parameters for Pickering emulsions

	Treatment	$\:{\boldsymbol{\sigma\:}}_{0}$ (Pa)	$\:\boldsymbol{\kappa\:}$ (Pa s^*n*^)	*n* (-)
Day 0	S13T2	25.07 ± 0.26^aA^	7.59 ± 0.13^aB^	0.514 ± 0.003^aB^
	S13T4	21.31 ± 0.22^aB^	6.59 ± 0.10^aC^	0.534 ± 0.003^aA^
	S15T2	25.03 ± 0.38^aA^	6.94 ± 0.18^aBC^	0.520 ± 0.005^aAB^
	S15T4	25.27 ± 0.84^aA^	9.11 ± 0.46^aA^	0.474 ± 0.009^aC^
Day 28	S13T2	30.60 ± 0.41^bA^	4.69 ± 0.17^bB^	0.583 ± 0.007^bA^
	S13T4	26.26 ± 0.33^bB^	5.00 ± 0.15^bAB^	0.544 ± 0.005^bC^
	S15T2	25.24 ± 0.31^aC^	5.09 ± 0.14^bA^	0.552 ± 0.005^bBC^
	S15T4	29.51 ± 0.31^bD^	4.89 ± 0.14^bAB^	0.560 ± 0.005^bC^

Means ± standard deviation (Tukey, *p* < 0.05)

Different capital letters indicate significant differences within the same storage time. Different lowercase letters indicate significant differences within the same treatment across storage times

At day 0, the S15T4 treatment exhibited the highest yield stress (25.26 Pa), while the S13T4 treatment was more fluid with $\:{\sigma\:}_{0}\:$= 21.31 Pa. For all the samples studied, the yield stress increased after 28 days of storage at 22%, 23%, 0.8%, and 17% for S13T2, S13T4, S15T2, and S15T4, respectively. However, the S15T2 emulsion exhibited the lowest yield stress after 28 days of storage with no statistically significant difference throughout storage time. Regarding the Herschel–Bulkley parameters, the flow behavior index (n) remained close to 0.5 at both day 0 and day 28 for all treatments, confirming the shear-thinning behavior characteristic of Pickering emulsions while a decrease in the consistency index ($\:\kappa\:$) was observed over storage time for all treatments, indicating a reduction in flow resistance, behavior may be attributed to structural rearrangements and improved droplet packing within the emulsion [99].

Similar values for Pickering emulsion formulations used in topical products were reported by Marto et al., [52], Marto et al., [54], Marto et al., [55] and Chiari-Andréo et al., [11], despite the use of different solid particles. Considering the particle size generated by each treatment, slight variations in particle size led to changes in the flow curves, with the smallest particles producing the lowest yield points and consistency values with the least change over time [55].

Despite the particle sizes generated being smaller (0.65–0.69 μm) than those reported by Marto et al., [54] for Pickering emulsions (5.54–8.87 μm) with green coffee oil stabilized with particles of TiO_**2**_, similar values and trends in flow curves are observed when using different types of solid particles. In this case, the similarity can be attributed to the type of emulsion, as factors such as concentration, particle interactions, and the formation of clusters of these particles increase viscosity and create a complex structure in the interfacial region, promoting greater stability, as previously reported [26, 60, 76].

After storage, all systems show a decrease in the shear stress required to generate fluid movement (Table 4). Those effects may result from structural changes in the emulsion packing, the formation of aligned structures between ZnO nanoparticles, and the presence of some texture modifiers such as xanthan gum [73, 90]. Interestingly, this decrease does not affect the emulsion’s spreadability, as the previously described behavior remains unchanged.

#### Oscillatory test (Frequency and amplitude sweep)

The amplitude sweep test of each emulsion shows that they have a linear viscoelastic region (LVR) between shear deformations of 0.01% to 0.1%,indicating the range of values where the moduli are independent of the applied deformation [61]. The strain γ = 0.05% was used in all frequency sweep tests based on preliminary amplitude sweep tests (data not shown). The emulsion frequency sweep curves showed that the elastic and viscous moduli varied minimally over the tested range.

**Fig. 7:**
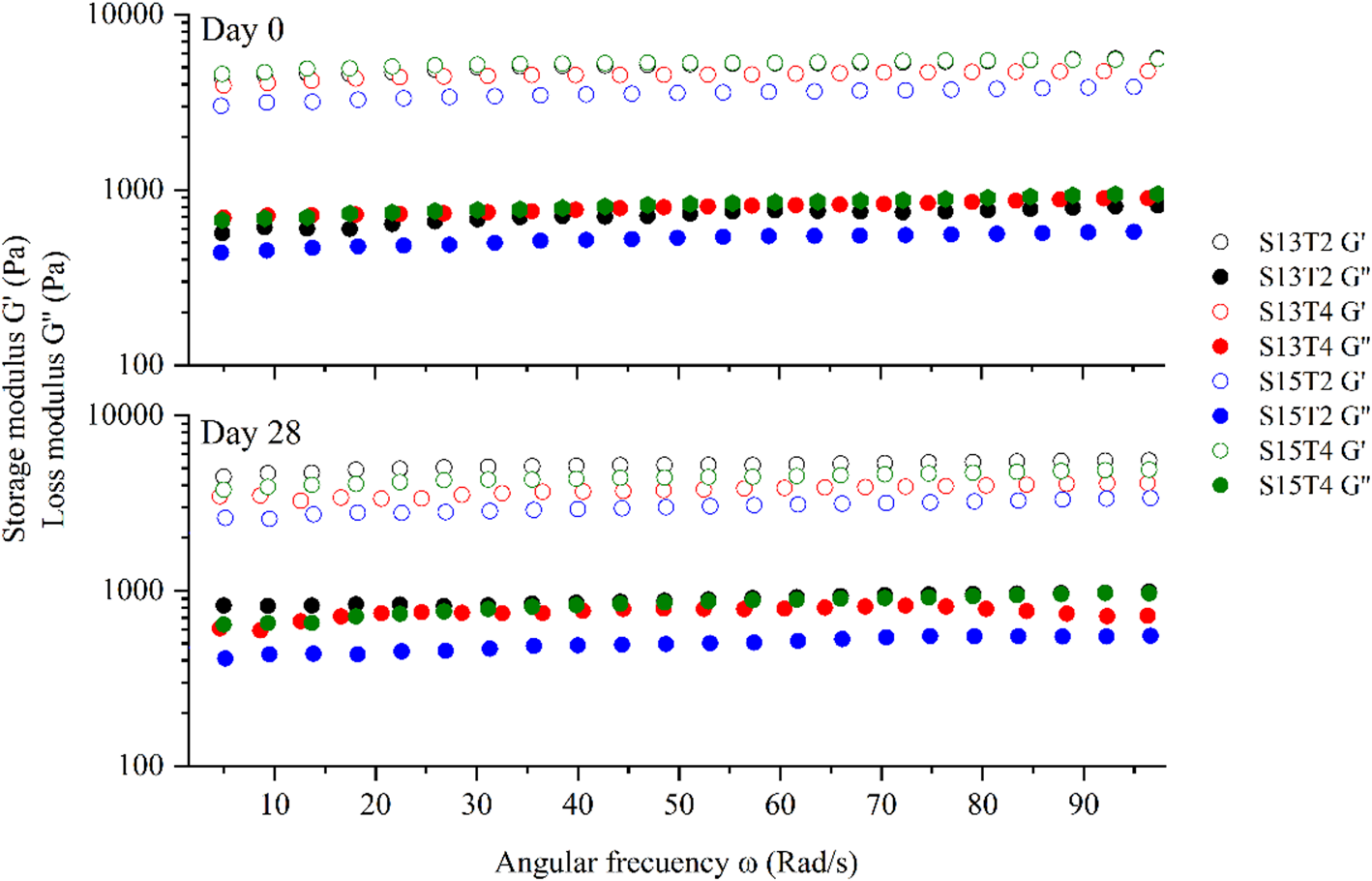
Frequency sweep (averages, *n* = 3) of emulsions. Predominance of $G^{\prime}$ over $G^{\prime\prime}$ indicates a gel-like behavior, influenced by particle interactions and structural rearrangements over time

In the frequency sweep tests, the moduli that make up the fluids can be observed (Fig. 7), where $G^{\prime}$ corresponds to the storage modulus or elastic part, and $G^{\prime\prime}$ indicates the loss modulus or viscous part. By analyzing the arrangement of both moduli and their values, it is possible to determine the behavior of the structure, its stability, and consistency [97]. According to Fig. 7, all treatments showed higher values of $G^{\prime}$ than $G^{\prime\prime}$, indicating a gel-like behavior in which the structure resists structural breakdown and exhibits a more solid consistency. Hohl et al., [38], Wei and Huang [96], and Xu et al., [98] associate this phenomenon with the presence of solid particles, which increase the viscosity of the system and form a structural network that prevents loss of plasticity and enhances its stability.

The frequency sweeps of the different systems showed a decrease in both moduli across all treatments (Fig. 7). Some authors, including Franco et al., [26], Isaac et al., [39], and Fazilati et al., [23], have attributed this decrease to a restructuring of the emulsion during this period due to the interaction between the phases and the particles, leading to a repacking of the system by rearranging the droplets in the emulsion. However, this repacking did not alter the flow behavior of the systems, so their ability to flow and be dispersed on the skin remained constant during storage.

### In vitro sun protection factor

The four emulsions containing 23.63% ZnO exhibited SPF values > 40, which is considered high protection by the European Commission [14]. Ahmad-Zaki et al., [2] reported SPF values ranging from 40.37 to 40.16 for sunscreens with similar ZnO nanoparticle concentrations, values comparable to those obtained for a commercial sunscreen with 25% ZnO labeled as 50 FPS (56.38 ± 1.96). These values can be attributed to the extensive contact area provided by the nanosized particles relative to their volume, resulting in greater optical interaction in the UVA and UVB regions. Other authors have also reported this behavior in sunscreens containing ZnO or TiO_**2**_ nanoparticles smaller than 100 nm [30, 80].

**Fig. 8:**
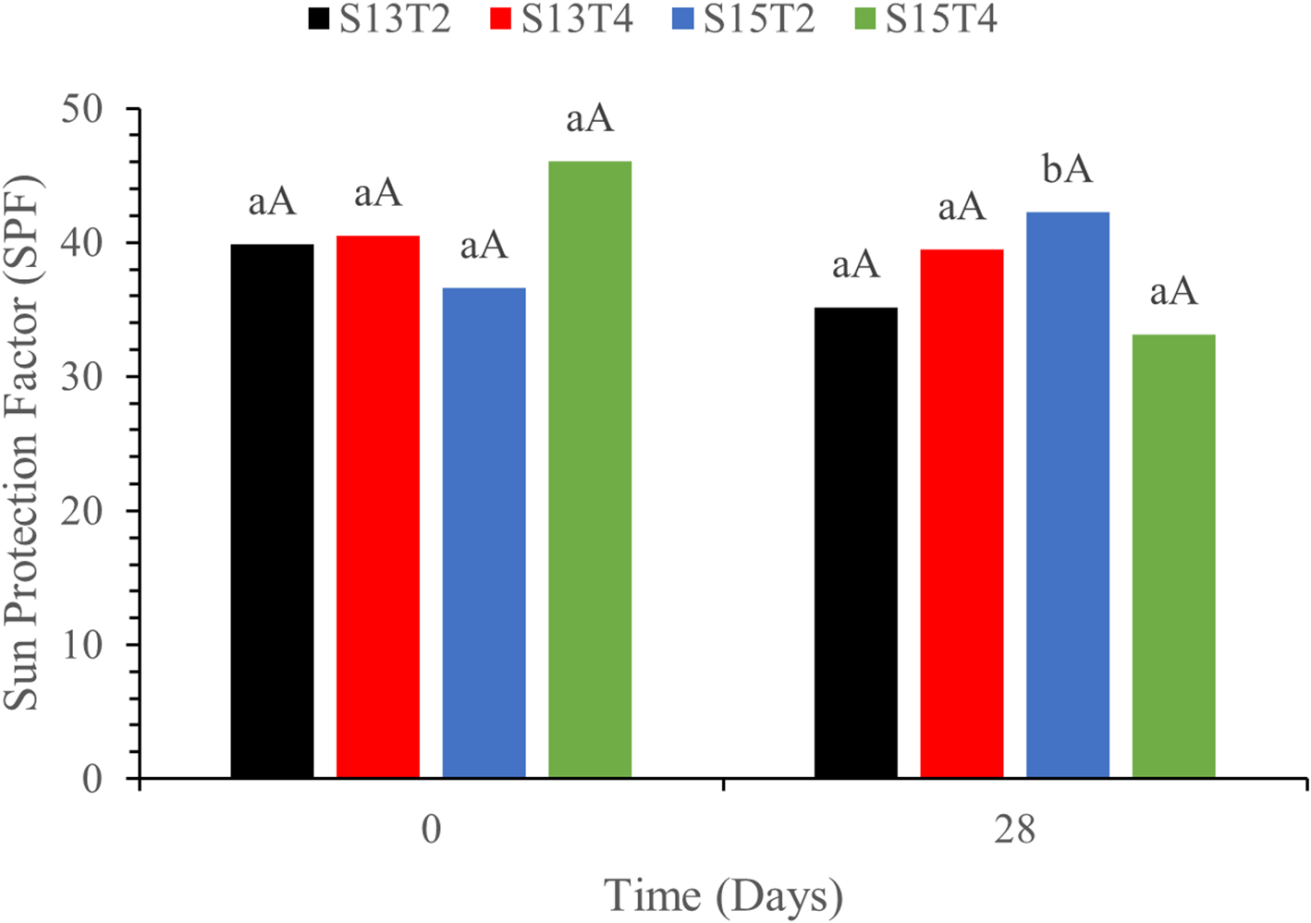
Sun protection factor of emulsions on days 0 and 28. (Tukey, *p* < 0.05). Lowercase letters compare the same treatment over storage time; uppercase letters compare all treatments at each condition. Equal letters indicate no statistically significant difference

SPF values were comparable across all emulsions (Fig. 8), suggesting that variations in time or processing conditions did not significantly affect the photoprotective capacity of each formulation. This performance can be attributed to the use of ZnO nanoparticles, since the optical properties of the physical barrier are the primary source of protection, providing optimized SPF values [10, 20, 80]. However, the operating conditions used in our formulations were inadequate to modify the SPF; therefore, further research is necessary to verify that these conditions do not affect this factor.

### Inhibition of ABTS^•+^ radicals

**Fig. 9:**
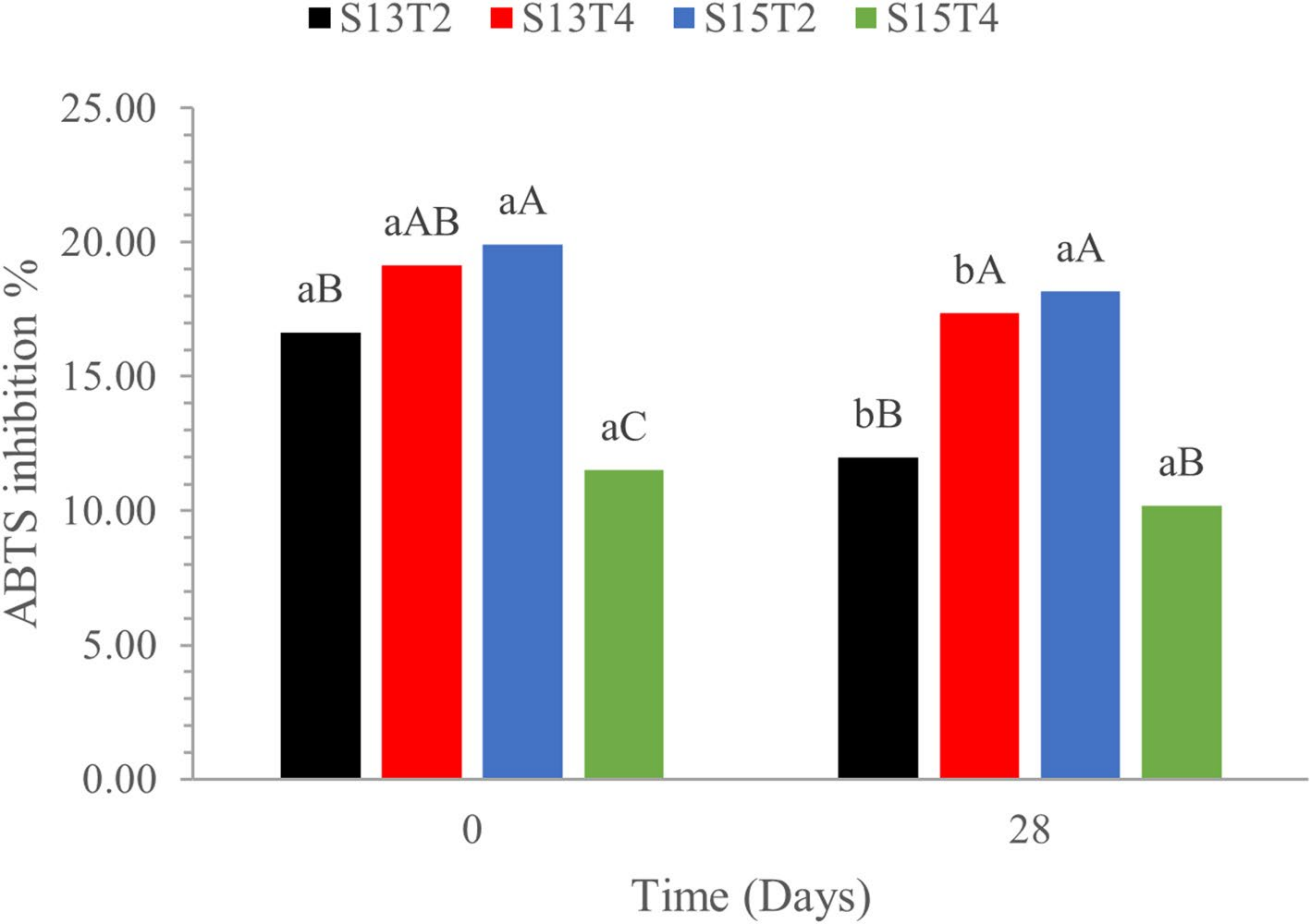
ABTS^**•+**^ Inhibition percentage of emulsions elaborated by different treatments on days 0 and 28. Lowercase letters compare the same treatment over time; uppercase letters compare different treatments at the same time. Means with the same letter are not significantly different (Tukey, *p* < 0.05)

Exposure to solar UV radiation facilitates the generation of free radicals, a factor that determines their harmful effects on human skin damage [18]. To evaluate the antioxidant potential of the emulsified systems, a spectroscopic assay was used to measure in vitro the neutralization of the radical cation ABTS^**•+**^. At time 0, all emulsions exhibited similar inhibition by 16–20%, except for the S15T4 emulsion, which had a significantly lower inhibition activity by 10% (*p* < 0.05). After storage (day 28), the ABTS^**•+**^ radical inhibition of S13T2 emulsion significantly decays to 12% (*p* < 0.05). On the other hand, S13T4 and S15T2 formulations display the same level of activities as day 0 (*p* > 0.05) (Fig. 9).

Since the emulsion is composed of various components, with quercetin serving as a standout antioxidant, changes in its activity could be related to potential impacts on the speeds and times applied during its preparation [36]. These potential effects include inadequate or uneven distribution of the wall material for encapsulation as well as an increase in temperature associated with the applied speeds, which could degrade the bioactive component [9, 88].

Figure 9 shows that treatment S13T2 had the greatest decrease in ABTS^**•+**^ radical inhibition. This behavior is attributed to poor encapsulation and the formation of a ZnO coating around the bioactive compound, influenced by the speed-time correlation [97, 102]. Conversely, treatment S15T4 showed the lowest inhibition rates, suggesting that increases in speed (rpm) and prolonged processing times may affect the antioxidant capacity of those systems. However, it is important to note that the emulsion obtained from the S15T4 system conserves its ABTS^•^^**+**^ radical inhibition activity after 28 days of storage.

In contrast, the S13T4 and S15T2 treatments did not show a statistically significant difference over time (*p* > 0.05), suggesting that the compound may provide effective protection and enhanced structural conformation stability. This behavior could be associated with the relationship between speed and time that incorporates energy into the system, suggesting remarkable chemical stability that preserves quercetin’s bioactivity.

For a quercetin concentration of 0.5 µg/mL, comparable to the one used in this study to evaluate antioxidant capacity using ABTS^**•+**^, previous studies, by Uzma et al., [93] and Chittasupho et al., [12], reported antioxidant activity values of ~ 10–15%. These values were lower than those obtained in this study for the S13T4 and S15T2 treatments, which were 19% and 20%, respectively. The observed increase could be attributed to a synergistic effect of quercetin and the antioxidant activity of the other components used in those emulsions. In this context, green coffee oil is known for its free radical inhibitory effects due to its chemical composition [94, 95]. Additionally, ZnO nanoparticles have been highlighted for their antioxidant properties, further enhancing free radical inhibition [19, 22, 66, 71].

## Conclusions

The Pickering emulsions studied in this work had particle sizes greater than 100 nm and SPF values of ~ 40, suggesting greater optical interaction in the UV-A and UV-B regions. These values were preserved throughout 28 days of storage, demonstrating the viability of these systems for use in pharmaceutical and cosmetical applications. Regarding the Turbiscan Stability Index, the results show that homogenization time influenced the physical stability of the emulsions, with the S13T4 and S15T2 systems being the most stable in terms of physicochemical properties. In particular, the S15T4 emulsion exhibited the lowest ABTS^**•+**^ inhibition values. Furthermore, over time, rheological properties showed shear-thinning behavior, with yield points within the preferred range for topical applications. Additionally, the HB model adequately described the flow behavior of the samples.

In conclusion, among the emulsions tested, S15T2 had the best physicochemical and viscoelastic properties. It showed a lower increase in particle size, maintained yield stress, and higher antioxidant capacity, and SPF values over 28 days of storage. This stability is associated with improved encapsulation and nanoparticle distribution at the interfacial region, which could be attributed to the structural integrity of each emulsion, making it a suitable vehicle for developing systems with great potential in pharmacology and cosmetology applications. At this point, the interactions between Pickering emulsions and the skin stratum layers remain to be determined to discard any associated toxicityof those formulations. It is also necessary to study bioactivity of quercetin in other encapsulated nanocarriers to evaluate its therapeutic potential for different skin conditions.

## Data Availability

The datasets generated and/or analyzed during the current study are available from the corresponding author on reasonable request.
